# Mechanisms of the gabapentinoids and *α*_2_ *δ*‐1 calcium channel subunit in neuropathic pain

**DOI:** 10.1002/prp2.205

**Published:** 2016-02-27

**Authors:** Ryan Patel, Anthony H. Dickenson

**Affiliations:** ^1^ Department of Neuroscience, Physiology and PharmacologyUniversity College LondonGower StreetLondonWC1E 6BTUK

**Keywords:** Alpha 2 delta 1, calcium channel subunit, gabapentin, neuropathic pain, pregabalin

## Abstract

The gabapentinoid drugs gabapentin and pregabalin are key front‐line therapies for various neuropathies of peripheral and central origin. Originally designed as analogs of GABA, the gabapentinoids bind to the *α*_2_ *δ*‐1 and *α*_2_ *δ*‐2 auxiliary subunits of calcium channels, though only the former has been implicated in the development of neuropathy in animal models. Transgenic approaches also identify *α*_2_ *δ*‐1 as key in mediating the analgesic effects of gabapentinoids, however the precise molecular mechanisms remain unclear. Here we review the current understanding of the pathophysiological role of the *α*_2_ *δ*‐1 subunit, the mechanisms of analgesic action of gabapentinoid drugs and implications for efficacy in the clinic. Despite widespread use, the number needed to treat for gabapentin and pregabalin averages from 3 to 8 across neuropathies. The failure to treat large numbers of patients adequately necessitates a novel approach to treatment selection. Stratifying patients by sensory profiles may imply common underlying mechanisms, and a greater understanding of these mechanisms could lead to more direct targeting of gabapentinoids.

**Abbreviations**
GBPs: gabapentinoids
DRG: dorsal root ganglia
NA: noradrenaline
GABA: *γ*‐aminobutyric acid
PAG: periaqueductal gray
Ce: central nucleus of the amygdala
H: hypothalamus
LC: locus coeruleus
IC: insula cortex
ACC: anterior cingulate cortex
RVM: rostral ventromedial medulla
VGCC: voltage gated calcium channel
SNL: spinal nerve ligation

## Introduction

The anticonvulsant gabapentin was first reported as providing pain relief 20 years ago (Mellick et al. 1995). The discovery that anticonvulsants could be used as analgesics started a scientific journey leading to the inclusion of gabapentinoids as key frontline therapy for various neuropathies. However, as with drugs of other classes, the NNT (number needed to treat) for gabapentinoids vary considerably between disease states (Finnerup et al. 2015). One of the perennial clinical questions is why do some patients respond positively to a treatment whereas others do not? Undoubtedly animal models have contributed immensely to our understanding of the neurobiology of pain. This is particularly evident with gabapentin where the back translation highlighted the permissive conditions for analgesic activity and the mechanisms that underpin these. Prior to the establishment of nerve injury models, drugs were commonly characterized using the rat paw formalin test, and Carrageenan‐induced mechanical hyperalgesia and thermal hyperalgesia since it was thought that enhanced pain processing was the key factor to understanding pain mechanisms without consideration of the very different peripheral drivers of pain from nerve and tissue damage. In addition, measures of thermal hyperalgesia and tactile allodynia in the rat postoperative pain model were employed. Although gabapentin shows efficacy in some of these models (Shimoyama et al. 1997; Stanfa et al. 1997), we now know that the effects of nerve injury and inflammation are very different and that these short‐term inflammatory events are not indicative of persistent inflammation such as arthritis for which models now exist. Rather, now and at the time, the non‐nerve injury models induced peripheral and central changes in pain processing that were thought to underlie the more persistent pains, independent of the causes. A key paper was Hunter et al. in 1997. The Hunter study was a landmark but very tellingly, the title is “The effect of novel anti‐epileptic drugs in rat experimental models of acute and chronic pain.” So even here, the concept was not that nerve injury necessarily would be different from other models but was of longer duration than the acute tests and that acute tests may not be predictive of persistent pain. They further state, “this ability to acutely reverse a prominent manifestation of neuronal sensitization demonstrates the potential of these drugs as analgesics for the relief of chronic pain following tissue or nerve injury. Moreover, the negligible effect of these drugs against an acute, high threshold thermal noxious stimulus suggests a selective interaction with pathways associated with pathophysiological events rather than with normal sensory nociceptive function.” Herein we review the current understanding of the state‐dependent mechanisms of the gabapentinoids, the pathophysiological role of their molecular target, the *α*_2_ *δ* calcium channel subunit, and the implications for clinical usage.

## α_2_δ Auxiliary Calcium Channel Subunits

**Figure 1:**
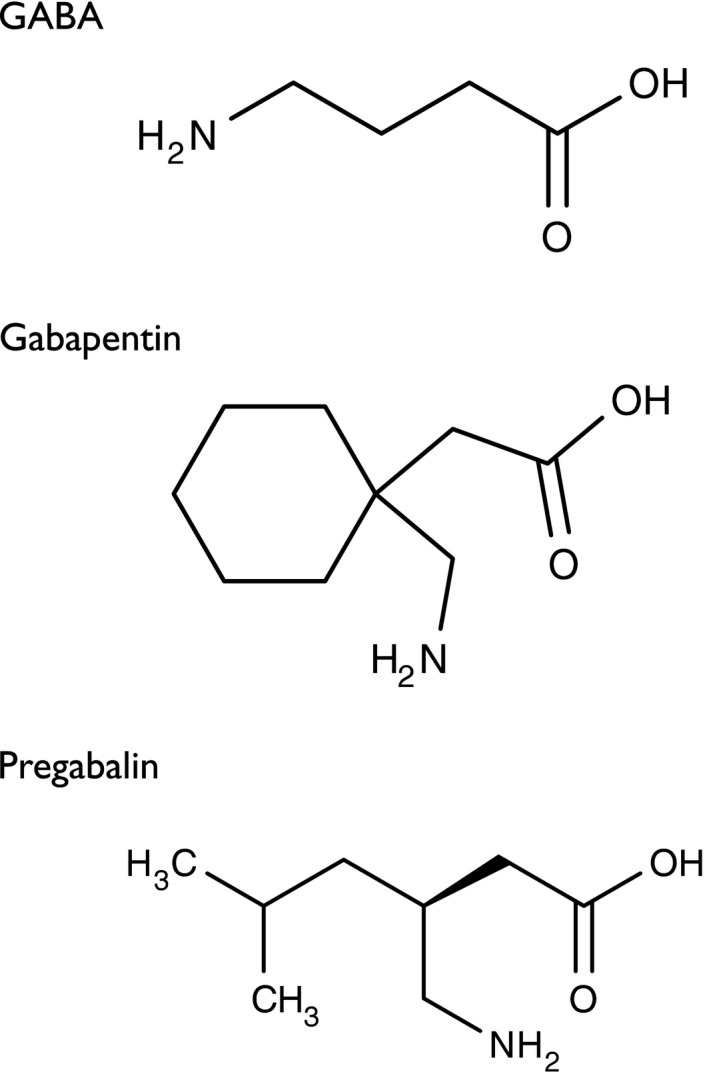
Structure of GABA, gabapentin and pregabalin.

**Figure 2:**
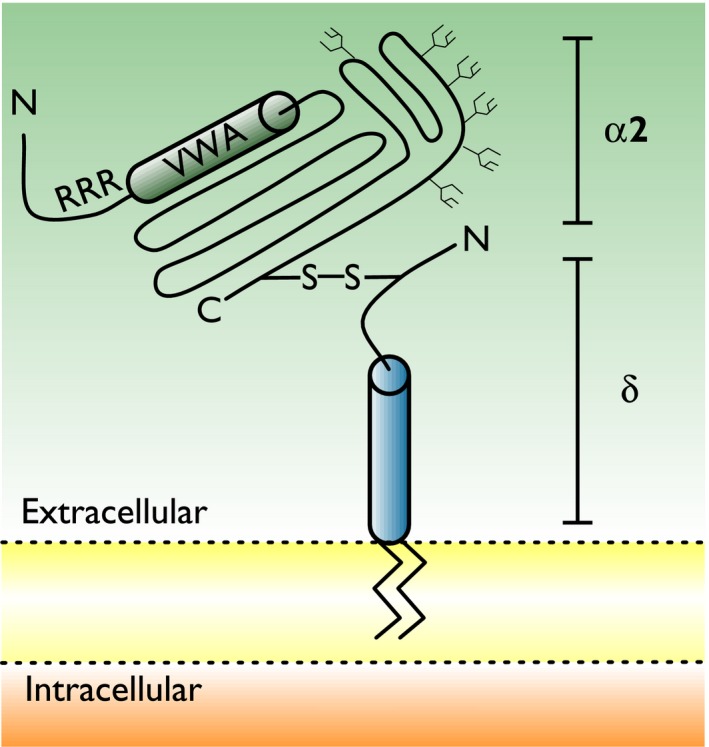
Topology of *α*_2_ *δ* subunits. All subunits are products of a single‐gene cleaved posttranslation and joined by disulphide bridges (De Jongh et al. 1990). The *δ* subunit is anchored to the plasma membrane through a glycosylphosphatidylinositol anchor (Davies et al. 2010). Putative N‐glycosylation sites have been identified on both domains. The approximate position of the von Willebrand factor A domain (required for trafficking of *α*_1_ subunit of VGCCs (Canti et al. 2005)) is shown in close proximity to gabapentin and pregabalin‐binding site in *α*_2_ *δ*‐1 and *α*_2_ *δ*‐2 (RRR).

Originally designed as analogs of GABA (Fig. 1), neither gabapentin nor pregabalin has any significant agonist‐like effect on GABA_A_ or GABA_B_ receptors, nor obvious effects on levels of GABA (Lanneau et al. 2001; Jensen et al. 2002). Gabapentin was discovered to bind to *α*_2_ *δ* subunits (Fig. 2) (Gee et al. 1996), with greater affinity for *α*_2_ *δ*‐1 (*K*_d_ = 59 nmol/L) compared to *α*_2_ *δ*‐2 (*K*_d_ = 153 nmol/L) and no affinity for *α*_2_ *δ*‐3 (Marais et al. 2001). The main biophysical and pharmacological properties of voltage gated calcium channels (VGCCs) are determined by the pore‐forming *α*_1_ subunit, whereas the *α*_2_ *δ*,* β* and *γ* components influence trafficking and activation kinetics (Arikkath and Campbell 2003). In vitro, *α*_2_ *δ* subunits indiscriminately associate with *α*_1_ subunits of VGCCs; the tissue selective expression of calcium channel components determine the composition and biophysical properties of heteromeric complexes in vivo (Dolphin 2013). In general, in heterologous expression systems, *α*_2_ *δ* subunits increase maximum current density and accelerate activation and inactivation of calcium currents (De Waard and Campbell 1995; Klugbauer et al. 1999; Hobom et al. 2000). The increase in current density is dependent on *α*_2_ *δ* subunits‐enhancing trafficking of *α*_1_ to the cell membrane rather than directly influencing channel kinetics (Felix et al. 1997). *α*_2_ *δ*‐1 may also stabilise calcium channels at the cell surface as its absence promotes internalization and degradation of channel complexes (Bernstein and Jones 2007), though this finding was not replicated in neuronal cultures (Cassidy et al. 2014).

Within the spinal cord, *α*_2_ *δ*‐1 is predominantly expressed presynaptically in the dorsal horn localized to the superficial laminae with moderate expression postsynaptically on deeper neurones (Li et al. 2006; Bauer et al. 2009). The presynaptic expression is highest in a subset of small diameter DRG neurones, predominantly C‐fibres, with moderate levels in a minor proportion of large diameter neurones (Taylor and Garrido 2008). *α*_2_ *δ* subunits are critical in trafficking of calcium channels to active zones of synapses, and controlling calcium influx and subsequent transmitter release (Hoppa et al. 2012). Within areas of the brain associated with nociceptive processing, moderate to strong expression has also been observed in the dorsal raphe, periaqueductal gray, locus coeruleus, and amygdala (Cole et al. 2005; Taylor and Garrido 2008). In the cerebellum, *α*_2_ *δ*‐2 subunits are concentrated within lipid raft microdomains (Davies et al. 2006). Lipid raft association has not been confirmed within the dorsal horn or in primary afferent fibres where *α*_2_ *δ*‐1 has been shown to be anterogradely transported following nerve ligation (Bauer et al. 2009), but the localization of *α*_2_ *δ* to lipid rafts could have consequences for nociceptive transduction as depletion of cholesterol enhances peak calcium currents (Davies et al. 2006).

## Role of α_2_δ‐1 in Normal Sensory Function and Experimental Neuropathy

*α*_2_ *δ*‐1 knockout mice were originally characterized as exhibiting reduced myocardial contractibility and decreased peak L‐type calcium current amplitude in cardiac myocytes (Fuller‐Bicer et al. 2009). Contrasting reports exist regarding whether *α*_2_ *δ*‐1 is required for muscle development (Gach et al. 2008; Garcia et al. 2008), however no obvious muscle weakness is apparent in the knockout mice (Fuller‐Bicer et al. 2009; Patel et al. 2013). Distension of the bladder occurs in a minor proportion of *α*_2_ *δ*‐1 knockout mice (Fuller‐Bicer et al. 2009), consistent with a role of the *α*_2_ *δ*‐1 subunit in the contractibility of smooth muscle cells (Bannister et al. 2009). Subsequent studies examined somatosensory functions; *α*_2_ *δ*‐1 knockout mice were shown to exhibit behavioral deficits in mechanical and cold sensitivity, but not heat sensitivity, a feature that corresponded with wide dynamic range (WDR) neuronal responses to the same stimuli (Patel et al. 2013). This likely relates to a reduction in trafficking of N‐type calcium channels to presynaptic terminals of primary sensory afferents in the dorsal horn (Patel et al. 2013).

After nerve injury, transcriptional alterations that occur are considered to be an adaptive response to preserve neuronal function. These changes can contribute to neuronal hyperexcitability and spinal plasticity in neuropathic pain. Numerous studies have identified an up‐regulation of *α*_2_ *δ*‐1 in DRG neurones (Luo et al. 2001; Costigan et al. 2002; Wang et al. 2002; Xiao et al. 2002) and the spinal cord (Boroujerdi et al. 2008; Bauer et al. 2009) after nerve injury, though supraspinal changes have not been examined in neuropathic models. Spinal nerve ligation (SNL) induces increased levels of *α*_2_ *δ*‐1 in presynaptic terminals of primary afferent fibres in the dorsal horn in addition to an accumulation proximal to the ligation indicative of anterograde trafficking (Bauer et al. 2009). Elevated DRG expression of *α*_2_ *δ*‐1 is readily detectable after SNL peaking 7 days after injury and declining after several months, a feature that temporally correlates with the emergence and cessation of neuropathic like evoked behaviors (Luo et al. 2001). A circadian fluctuation in DRG *α*_2_ *δ*‐1 expression also correlates with changes in behavioral hypersensitivity during light and dark phases (Kusunose et al. 2010). Established mechanical hypersensitivity can be reversed by intrathecal antisense oligonucleotides and prevented by dorsal rhizotomy at the time of nerve ligation (Li et al. 2004). *α*_2_ *δ*‐1 knockout mice, however, exhibit a delay in developing mechanical hypersensitivity following nerve injury. In the early parts of the model, *α*_2_ *δ*‐1 expression appears to be a rate‐limiting factor in transmitting abnormal peripheral activity to central neurones and is key in shaping the initiation of neuropathic pain, but the absence of *α*_2_ *δ*‐1 fails to prevent chronicity and is not essential in the maintenance of a neuropathic state (Patel et al. 2013).

Some of the mechanisms by which increased *α*_2_ *δ*‐1 expression facilitates excitatory transmission in neuropathic animals have been examined in transgenic mice overexpressing *α*_2_ *δ*‐1. In the absence of injury, these transgenic mice have mechanical withdrawal thresholds comparable to nerve ligated wildtype controls (Li et al. 2006), and this may in part be dependent on increased *α*_2_ *δ*‐1 mediated trafficking of L‐ and N‐type calcium channels to the dorsal horn (Chang et al. 2015). DRG neurones from transgenic mice exhibit a hyperpolarizing shift in the voltage activation of VGCCs, increased peak conductance and an increased inactivation (Li et al. 2006). Deep dorsal horn WDR neurones in transgenic mice exhibit responses to low threshold mechanical and heat stimulation of the receptive field greater than high threshold stimuli in wild‐type mice. A pronounced and prolonged after‐firing characteristic of central neuronal hyperexcitability is also notable (Li et al. 2006), though electrically evoked wind‐up is unaltered in transgenic mice suggesting no change in intrinsic WDR excitability (Li et al. 2006; Nguyen et al. 2009). Overexpression of *α*_2_ *δ*‐1 increases the frequency, but not the amplitude, of mEPSCs in the dorsal horn and is reversed by gabapentin, and inhibition of AMPA and NMDA receptors (Nguyen et al. 2009; Zhou and Luo 2014). These data support an increase in afferent excitability and subsequent spinal neuronal responses in mediating behavioral abnormalities in *α*_2_ *δ*‐1 overexpressing mice. Interestingly, the increase in mechanical withdrawal thresholds observed in *α*_2_ *δ*‐1 overexpressing mice following intrathecal ondansetron (a 5‐HT_3_R antagonist) bears marked similarities to the inhibitory effect of ondansetron on mechanically evoked neuronal responses in SNL rats suggesting overexpression of *α*_2_ *δ*‐1 in the absence of injury is sufficient to drive changes in descending serotonergic facilitations which terminate on these spinally expressed receptors (Suzuki et al. 2004; Chang et al. 2013).

## Mechanisms of Gabapentinoid Activity after Neuropathic Injury

**Figure 3:**
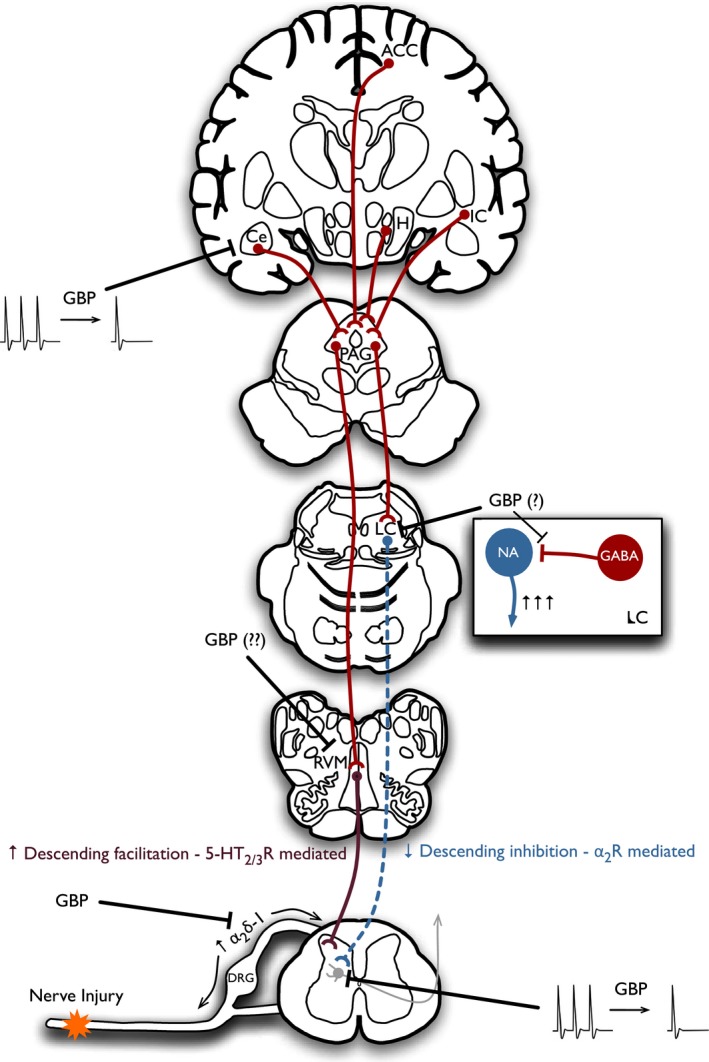
Sites of action of gabapentinoid drugs. Nerve injury induces synaptic plasticity, central sensitization, increased descending serotonergic facilitation and reduced descending noradrenergic inhibition of spinal neuronal excitability. Gabapentinoid drugs have been demonstrated to inhibit trafficking of the *α*_2_ *δ*‐1 subunit from DRG neurones to central terminals in the dorsal horn. Spinally administered gabapentinoids in neuropathic rats reduce dorsal horn neuronal responses to peripheral stimuli. This effect is highly influenced by an increase in descending serotonergic facilitations. Gabapentinoids have also been proposed to restore deficiencies of descending noradrenergic inhibition, although the spinal effects do not depend on this pathway. Gabapentinoids reduce elevated spontaneous and evoked activity in the amygdala, though this may occur secondary to reduced spinal neuronal activity. (GBPs, gabapentinoids; DRG, dorsal root ganglia; NA, noradrenaline; PAG, periaqueductal gray; Ce, central nucleus of the amygdala; H, hypothalamus; LC, locus coeruleus; IC, insula cortex; ACC, anterior cingulate cortex; RVM, rostral ventromedial medulla).

The mechanism of action of gabapentinoids at the cellular level and after neuropathy has been the subject of much debate. Can a single molecular mechanism explain all aspects of analgesia? Point mutation of arginine 217 in *α*_2_ *δ*‐1 or genetic ablation of *α*_2_ *δ*‐1 completely abolishes the antinociceptive effects of pregabalin in neuropathic mice (Field et al. 2006; Patel et al. 2013). Several mechanisms of gabapentin have been proposed after neuropathy including an inhibition of NMDA receptors, inhibition of sodium currents and reducing *β*4a subunit mediated VGCC trafficking (Hara and Sata 2007; Mich and Horne 2008; Yang et al. 2009). Nevertheless, molecular and transgenic studies strongly support *α*_2_ *δ*‐1 as the sole molecular target for the analgesic actions of gabapentinoid drugs (summarized in Fig. 3).

Numerous studies confirm that gabapentinoids do not perturb normal detection and pain thresholds (Attal et al. 1998; Dirks et al. 2002); the pathophysiological state‐dependent effects of pregabalin and gabapentin implies other factors influence efficacy in neuropathic conditions. *α*_2_ *δ* subunits have been implicated in mediating excitatory synapse formation through astrocyte‐derived thrombospondins independent from their role with calcium channels and is a process that is sensitive to gabapentin inhibition (Eroglu et al. 2009). It is unclear as to whether the analgesic effect of gabapentinoids is in part dependent on inhibiting new synapse formation, however this seems somewhat unlikely given that these agents are efficacious long after the development of neuropathy when presumably synaptic sprouting has already occurred.

Gabapentin and pregabalin are thought to inhibit transmitter release, though the precise molecular mechanism is currently undefined. The most plausible explanation would be a direct inhibition of VGCCs, yet calcium currents are not consistently reduced by acute gabapentin (Stefani et al. 1998; Sutton et al. 2002; Hendrich et al. 2008), whereas chronically applied gabapentin can reduce P‐type and N‐type calcium currents (Hendrich et al. 2008). Similarly in cultured tissue slices, gabapentin does not consistently inhibit transmitter release (Fink et al. 2000; Fehrenbacher et al. 2003; Brown and Randall 2005; Quintero et al. 2011). These findings are perhaps unsurprising given they were performed in normal tissue and the analgesic effects of gabapentinoids are mainly revealed by neuropathy. After up‐regulation in DRG neurones, the *α*_2_ *δ*‐1 subunit can influence activity‐dependent calcium signaling that could affect signaling cascades related to aberrant neurotransmission (D'Arco et al. 2015). An additional hypothesis proposed is that gabapentinoids could reduce neuronal hyperexcitability by modulation of these pathways.

**Figure 4:**
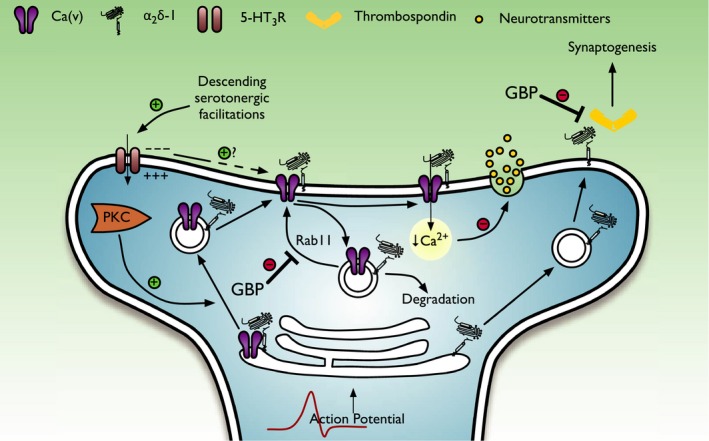
Cellular mechanisms of gabapentinoid drugs in the dorsal horn. *α*_2_ *δ*‐1 and *β* subunits of VGCCs mediate forward trafficking of channels from the endoplasmic reticulum, and this process can be facilitated by PKC. Descending facilitations activating presynaptic ionotropic 5‐HT_3_Rs results in membrane depolarization and may have consequences for VGCC activity. Gabapentinoids inhibit rab11‐dependent recycling of endosomal VGCCs while having no effect on the interaction between *α*_2_ *δ*‐1 and VGCCs at the membrane. An inhibition of channel recycling results in reduced channel expression at the synaptic membrane and a decrease in transmitter release. Independent of its association with VGCCs, *α*_2_ *δ*‐1 can interact with extracellular matrix proteins such as thrombospondins and mediate excitatory synaptogenesis, a process that is also inhibited by gabapentin. (GBPs, gabapentinoids; PKC, protein kinase C; VGCCs, voltage‐gated calcium channels).

Chronic systemic pregabalin treatment in SNL rats inhibits trafficking of *α*_2_ *δ*‐1 to presynaptic terminals in the dorsal horn (Bauer et al. 2009). Whether this mechanism would account for the acute effects of pregabalin in models of neuropathy seems unlikely given the time scale of axonal trafficking (Hunter et al. 1997; Field et al. 2006; Miyazaki and Yamamoto 2012; Patel et al. 2013). At the cell surface gabapentin does not disrupt the interaction between *α*_2_ *δ*‐1 and *α*_1B_ subunits (Cassidy et al. 2014). Gabapentin also fails to inhibit the internalization rate of *α*_2_ *δ*‐2 but does disrupt rab11‐dependent recycling from endosomal compartments consequently reducing calcium currents through this mechanism (Tran‐Van‐Minh and Dolphin 2010). It is tempting to speculate that at the spinal level, acute pregabalin and gabapentin treatment preferentially targets channel cycling pathways (Fig. 4), the rate of which may be influenced by multiple convergent factors governing pre‐synaptic excitability after neuropathy. One candidate is PKC, as the up‐regulation has been implicated in the development of neuropathy (Hua et al. 1999) and gabapentin inhibits transmitter release only after PKC‐dependent phosphorylation within spinal circuits (Maneuf and McKnight 2001; Fehrenbacher et al. 2003).

In neuropathy, permissible conditions for the inhibitory actions of spinally delivered gabapentinoids also depend on interactions between *α*_2_ *δ*‐1 and descending brainstem facilitations terminating on spinal 5‐HT_3_Rs. Targeted saporin conjugate disruption of a spino‐bulbal‐spinal loop comprising spinal NK1+ projection neurones, or *μ*‐opioid receptor‐expressing neurones in the rostral ventromedial medulla (RVM) negates gabapentin and pregabalin mediated inhibition of spinal neuronal excitability in neuropathic rats (Suzuki et al. 2005; Bee and Dickenson 2008). Remarkably, in naïve rats, mimicking increased facilitatory drive by applying a 5‐HT_3_R agonist spinally now induces a state permissible for the inhibitory actions of pregabalin (Suzuki et al. 2005). The depolarizing effect of activating pre‐synaptic 5‐HT_3_Rs could consequently alter the kinetics and/or cycling of calcium channels and create the required conditions for gabapentin to inhibit calcium currents and transmitter release (Suzuki et al. 2005). In contrast the conditions for the inhibitory effects of pregabalin in visceral hyperalgesia are independent of *μ*‐opioid receptor‐positive neurones of the RVM (Sikandar et al. 2012), and reflects differential brainstem control of cutanueous and visceral stimulation (Sikandar and Dickenson 2011).

Central sensitization and the subsequent engagement of descending influences, in some cases in the absence of pathology, is a key determinant of gabapentinoid analgesia and is evident in both humans and preclinical models. Neither gabapentin nor pregabalin inhibit acute nociceptive reflex responses in uninjured rodents (Field et al. 1997). In contrast, both drugs frequently display inhibitory activity in models with features of central sensitization such as after nerve ligation, but also in models where neuropathy is not the only component such as osteoarthritis and cancer‐induced bone pain or completely absent such as with opioid‐induced hyperalgesia (Donovan‐Rodriguez et al. 2005; Suzuki et al. 2005; Bannister et al. 2011; Thakur et al. 2012). In the case of opioid‐induced hyperalgesia, pregabalin suppresses spinal neuronal hyperexcitability in the absence of both pathology and up‐regulation of *α*_2_ *δ*‐1 (Bannister et al. 2011). Here, as after neuropathy, negating the effects of descending facilitation results in the loss of pregabalin efficacy, supporting that increased descending facilitation sustains spinal neuronal hyperexcitability in opioid‐induced hyperalgesia and that this mechanism underpins the state‐dependent inhibitory actions of pregabalin (Bannister et al. 2011). In healthy human volunteers, topically applied capsaicin is the archetypal model of central sensitization characterized by ongoing afferent activity leading to primary and secondary hyperalgesia and increased descending facilitatory drive (O'Neill et al. 2012). Both acute and chronic gabapentin are analgesic in capsaicin‐induced hyperalgesia and can reduce areas of secondary hyperalgesia (Gottrup et al. 2004; Iannetti et al. 2005). Brainstem activity detected by functional MRI is consistent with increased descending facilitatory drive in this model (Iannetti et al. 2005). A link between these descending facilitations and the actions of the gabapentinoids could be the targeting of TRPV1 (so pain transmitting) central terminals by 5‐HT_3_ facilitations (Kim et al. 2014); this sensitization could permit the actions of the drugs through *α*_2_ *δ*‐1 actions on non‐neuropathic pains without up‐regulation of the subunit.

Both intrathecally and intracerebroventricularly delivered gabapentin are efficacious after peripheral nerve injury (Tanabe et al. 2005; Hayashida et al. 2007; Takeuchi et al. 2007), the effect of the latter in part hypothesized to result from a disinhibition of locus coeruleus neurones promoting release of noradrenaline in the dorsal horn and is reversible by intrathecal *α*_2_ receptor antagonists (Tanabe et al. 2005; Takasu et al. 2008). Systemic pregabalin has also been shown to normalise elevated spontaneous and evoked neuronal activity in the central nucleus of the amygdala (Ce) in neuropathic states (Goncalves and Dickenson 2012). This effect may occur directly within the Ce or secondary to a reduction in spinal neuronal activity and in turn influence inhibitory and facilitatory output through the periaqueductal gray, locus coeruleus and RVM. Anxiolytic‐like activity of pregabalin is dependent on *α*_2_ *δ*‐1 but not *α*_2_ *δ*‐2 (Lotarski et al. 2011), and elevated expression of *α*_2_ *δ*‐1 in the amygdala correlates with increased predator odour‐induced anxiety and can be reversed by pregabalin (Nasca et al. 2013). Pregabalin displays an anxiolytic effect in patients (Feltner et al. 2003), and is now licenced for generalized anxiety disorders, and could indirectly influence pain perception through ascending or descending pathways.

## Implications for Clinical Efficacy

Going forward, it is important to consider the factors that may predict gabapentinoid efficacy and how to tailor treatment for individual patients. From this account, it is clear that a number of neurobiological events in pain contribute to the permissive neuronal milieu needed for actions of the drugs on the *α*_2_ *δ*‐1 subunits. In practice, it would be difficult to infer underlying mechanisms from symptoms in a patient and therefore drug efficacy, but there is an ever‐increasing body of evidence that subgrouping patients may be beneficial to predicting drug efficacy and this approach could be considered when prescribing gabapentinoids. Insights from animal studies identify up‐regulation of the *α*_2_ *δ*‐1 subunit as a key factor for gabapentinoid analgesia (Luo et al. 2001; Bauer et al. 2009). Interestingly, *α*_2_ *δ*‐1 splice variants exert similar effects on calcium currents, however after spinal nerve ligation variants are differentially expressed, in particular a variant with lower affinity for gabapentin and it is possible in some patients that expressional differences account for some of the variation in efficacy observed clinically (Lana et al. 2014). Single doses of gabapentinoids can alleviate ongoing pain and evoked pain (Berry and Petersen 2005), on much shorter times scales than required for axonal trafficking; clearly mechanisms independent of inhibiting trafficking are also important and here we have discussed the descending controls in this context.

A number of clinical trials suggest peri‐operative gabapentinoids provide a small reduction in postoperative pain and opioid consumption (Schmidt et al. 2013). Meta‐analysis of studies evaluating the effect of peri‐operative gabapentinoids have been complicated by comparing differing surgical procedures with varying propensity to developing chronic pain and further limited by low statistical power, variations in dosing regimes and a lack of long‐term follow up studies (>6 months). These longer follow up studies do not consistently demonstrate reduced incidences of neuropathic pain. Drawing parallels with the delayed development of neuropathy in *α*_2_ *δ*‐1 knockout mice, treatment at the time of injury might not necessarily prevent chronicity. In contrast, in established neuropathy, meta‐analysis supports that gabapentin and pregabalin display efficacy in various neuropathies of peripheral and central origin (Moore et al. 2009, 2014; Wiffen et al. 2013). The largest of these studies have been performed in postherpetic neuralgia and diabetic neuropathy, as well as fibromyalgia, which is not a neuropathic state. This efficacy in patients with nerve injury has lead to the proposal that osteoarthritis patients with neuropathic features would also be likely to benefit from gabapentinoid therapy (Thakur et al. 2014). Here preclinical studies reveal an action of the drugs in osteoarthritis models where there are neuropathic components and an up‐regulation of *α*_2_ *δ*‐1 levels in ipsilateral L3 and L4 DRG as well as induction of descending facilitations but not in “pure” osteoarthritis where these changes are absent (Rahman et al. 2009; Thakur et al. 2012). Though to date, only one clinical trial has demonstrated the potential benefit of combination therapy in osteoarthritis with pregabalin and meloxicam (Ohtori et al. 2013).

Ongoing pain scores are typically used as primary endpoints of efficacy in clinical trials complicating direct comparisons with animal studies where evoked stimuli are most often used, but in neuropathic rats gabapentin produces conditioned place preference, an indicator of reward and relief from ongoing pain (Griggs et al. 2015), and reduces spontaneous spinal neuronal activity, a possible neuronal correlate of ongoing pain (Suzuki and Dickenson 2006). Secondary outcome measures such as sleep and anxiety have shown improvement in some trials (Dworkin et al. 2003; van Seventer et al. 2010; Kim et al. 2011). Reports of negative trials involving gabapentin and pregabalin have also emerged and could be caused by the heterogeneity of patient groups rather than ineffectiveness of the drugs *per se* (Simpson et al. 2010; Kim et al. 2011). *Post hoc* analysis of baseline patient data from these trials supports that no sensory profile is associated with a particular etiology and identifies distinct transetiological clusters (Freeman et al. 2014). Overall pregabalin was not superior to placebo treatment in a trial involving HIV neuropathy patients; retrospective analysis however revealed pregabalin was more effective in subgroups exhibiting pinprick hyperalgesia (Simpson et al. 2010). Similarly in human experimental models of pain, gabapentin and pregabalin are effective against secondary pinprick hyperalgesia (Werner et al. 2001; Dirks et al. 2002; Segerdahl 2006; Chizh et al. 2007) and are particularly effective on mechanical responses of spinal cord neurones in animals. Although there are conflicting reports regarding the efficacy of gabapentinoids in inflammatory and neuropathic models against mechanical and thermal hypersensitivity, in neuropathic animals at least, these agents preferentially inhibit spinal neuronal responses to punctate and dynamic mechanical stimulation compared to thermal stimulation (Donovan‐Rodriguez et al. 2005; Bee and Dickenson 2008; Thakur et al. 2012). This is also supported by some clinical observations that heat pain thresholds are not frequently increased by gabapentinoids whereas mechanical pain thresholds are (Attal et al. 1998; Werner et al. 2001). Gabapentinoids can be efficacious against allodynia, hyperalgesia and ongoing pain in facilitated states independent of etiology. Variations in efficacy could also be shaped by activity within descending modulatory pathways and these circuits may be directly modulated by gabapentinoids.

Conditioned pain modulation (CPM) in healthy volunteers and patients provides a readout of the efficiency of descending inhibitory output, and has been utilized as a predictive tool for drug efficacy. Both human and animal studies demonstrate that CPM or diffuse noxious inhibitory controls (DNIC) are reduced or absent in neuropathic conditions, that reducing the effect of descending facilitation or enhancing inhibitions reveal DNIC in neuropathic rats, and low CPM predicts efficacy of duloxetine (SNRI) and tapentadol (MOR‐NRI) in diabetic neuropathy patients (Yarnitsky et al. 2012; Niesters et al. 2014; Bannister et al. 2015). Thus it could be expected that low CPM would be predictive of efficacy of any drugs that restore imbalances between descending inhibitory and excitatory influences. Although gabapentinoids have been suggested to act supraspinally in the locus coeruleus (Tanabe et al. 2005; Takasu et al. 2008), the only human study to date demonstrates low CPM does not predict pregabalin efficacy in pancreatitis patients with widespread cutaneous sensitization (Olesen et al. 2013), inconsistent with a role in enhancing descending noradrenergic inhibitory drive. Functional MRI analysis reveals gabapentin reduces brainstem activation induced by cutaneous capsaicin sensitization (Iannetti et al. 2005), but this could occur secondary to its spinally mediated effects rather than direct actions within the brainstem. Further studies are required to examine the correlation between gabapentinoids and CPM in other conditions but temporal summation also warrants further investigation. Temporal summation is frequently enhanced in neuropathic conditions and is seen as a proxy for central sensitization. Drugs shown to reduce central sensitization also inhibit temporal summation including ketamine and gabapentin (Koppert et al. 2001; Arendt‐Nielsen et al. 2007).

## Conclusion

Numerous lines of pre‐clinical and clinical evidence support that gabapentinoids can be useful treatments in conditions where features of central sensitisation are present, in particular secondary pinprick hyperalgesia. Animal studies unequivocally demonstrate that the α2δ‐1 calcium subunit controls transmitter release and up‐regulation further facilitates excitatory transmission in neuropathic conditions, and that the interaction of gabapentinoids with α2δ‐1 is necessary for the analgesic actions. At the cellular and molecular level, multiple mechanisms could account for the acute and chronic effects of dosing in neuropathic patients. Adverse effects associated with gabapentinoids, including somnolence, dizziness and ataxia, occur in healthy volunteers implying an ability to modulate transmitter release within the CNS. Furthermore, de novo sensitivity of those with chronic pain, epilepsy and anxiety disorders to gabapentinoids implicates other factors within CNS circuits that determine the additional inhibitory activity of these drugs. The comparative effects of gabapentinoids in animal models and neuropathic patients are suggestive of similar processes being involved. It is worth considering continuation of profiling and sub‐grouping of patients within trials as this could lead to the identification of better predictors of efficacy.

## Disclosures

None declared.
